# Malignant neoplasms of Meckel's diverticulum; an evidence based review

**DOI:** 10.1016/j.amsu.2019.05.017

**Published:** 2019-06-04

**Authors:** S.A. Kabir, S.A. Raza, S.I. Kabir

**Affiliations:** a University Hospital Coventry & Warwickshire, Clifford Bridge Road, Coventry CV2 2DX, UK; b Queen Elizabeth Hospital, University Hospitals Birmingham NHS Foundation Trust, B15 2TH, UK

**Keywords:** Meckel's diverticulum (MD) and malignancy, Neoplasms, Tumour, Carcinoid, Sarcoma, Adeno-carcinoma, Gastro intestinal stromal tumours and rare tumour

## Abstract

An up to date published literature has shown that Meckel's Diverticulum (MD) are discovered incidentally and are benign, malignant transformation is unusual with reported incidence to be only 0.5%–3.2%.

The research available on this rare tumour remains scanty, mainly consisting of case reports and case series with many researchers reporting on their own clinical experience and often disagree on not only its epidemiology, but also more so on its surgical indications. In addition to the above there is no agreed standard formal grading and staging classification for primary MD tumour that can not only help assess the tumour in a systematic way, but also advise on a standard treatment plan that is to be followed after emergency surgery.

Hence, the aim of this article is to systematically review the latest evidence on these rare types of malignant neoplasm originating from MD, and conclude the best management options when encountered with such situations.

## Highlights

• Primary malignancies of the MD are extremely rare with reported incidence to be only 0.5%–3.2%.
• The prognosis is significantly worse and surgical resection futile if the primary malignant tumour remains covert or residual disease is present after surgery.
• Adjuvant chemotherapy used appears to have no significant effect on its overall survival prognosis.
• At present there is no agreed standard formal grading and staging classification for primary MD tumour, that now needs to be developed.

## 1 Introduction

Meckel's Diverticulum (MD) was first reported in 1598 by a German surgeon named Fabricius Hildanus who observed a diverticulum occurring in the distal part of the ileum. It was not until 1809 that anatomist Johann Friedrich Meckel from Germany described its embryologic origin from the omphalo-mesenteric duct and hence it was named after him [1].

It is a true congenital diverticulum, which implies that it is derived from all layers of the small bowel from the mucosa up to the serosal layer. The blood supply is from a remnant of the vitelline artery that originates from the superior mesenteric artery, and follows the rule of 2's i.e. it affects 2% of the population, 2% of patients are symptomatic, it is mostly found 2 feet from the ileo-cecal valve, symptoms normally become evident before the age of 2 years, ectopic tissue can be found in 1 out of 2 cases, most are about 2 inches long and the ratio of male-to-female incidence is 2 to 1 [2].

Published Literature concludes that most of the MD are discovered incidentally and are benign, malignant transformation is unusual with reported incidence to be only 0.5%–3.2% [3,4]. Out of which Carcinoid is the most common primary diverticular malignancy 33%–44%, followed by Leiomyosarcoma 18%–25%, Adenocarcinoma 12%–16% and Gastro Intestinal Stromal Tumours (GISTs) representing 12% of primary tumours in MD. Rare histological sub-types include pancreatic carcinoma, intra ductal papillary mucinous neoplasms, lymphomas and melanomas[[5], [6], [7], [8], [9], [10], [11], [12], [13]].

The literature available on these rare histological sub –types remains scanty which mainly consist of case reports and case series with many researchers reporting on their own clinical experience and often disagree on not only its epidemiology, but more so on its surgical indications. The management to be followed in the case of malignant neoplasm of MD in adults is not yet unanimous [14,15].

The aim of this article is to systematically review the latest evidence on these rare types of malignant neoplasm originating from MD, and conclude the best way forward in these situations.

## 2 Methodology

A literature search was performed using multiple electronic search engines: PUBMED, MEDLINE, EMBASE and the Cochrane Database from January 2000 until October 2017.

The key search word and phrases that were used in this study were Meckel's Diverticulum (MD) and Malignancy; Neoplasms, Tumour, Carcinoid, sarcoma, Adeno-carcinoma, Gastro intestinal stromal tumours and rare tumour. They were used in mixed combinations to generate the utmost number of articles.

The references of the articles were also screened and included if deemed relevant. The data was gathered categorically for author of the study, date of publication, study design and clinical parameters assessed. Commonly used variables reviewed were clinical symptoms, investigations and imaging.

Inclusion and exclusion criteria that were applied:

1. The study included conclusive diagnoses of tumours related to MD.
2. Inclusion of at least one of our outcome measures mentioned above.
3. Studies of only human subjects.
4. Publication literature was English.

Our literature search revealed a final 3690 articles. Two independent researchers (SAK, SIK) screened title and abstracts, 3222 articles were considered irrelevant. A third independent reviewer (RS) reviewed equivocal cases. After applying inclusion and exclusion criteria, a total of 58 studies were selected for our final review.

**Fig. 1:**
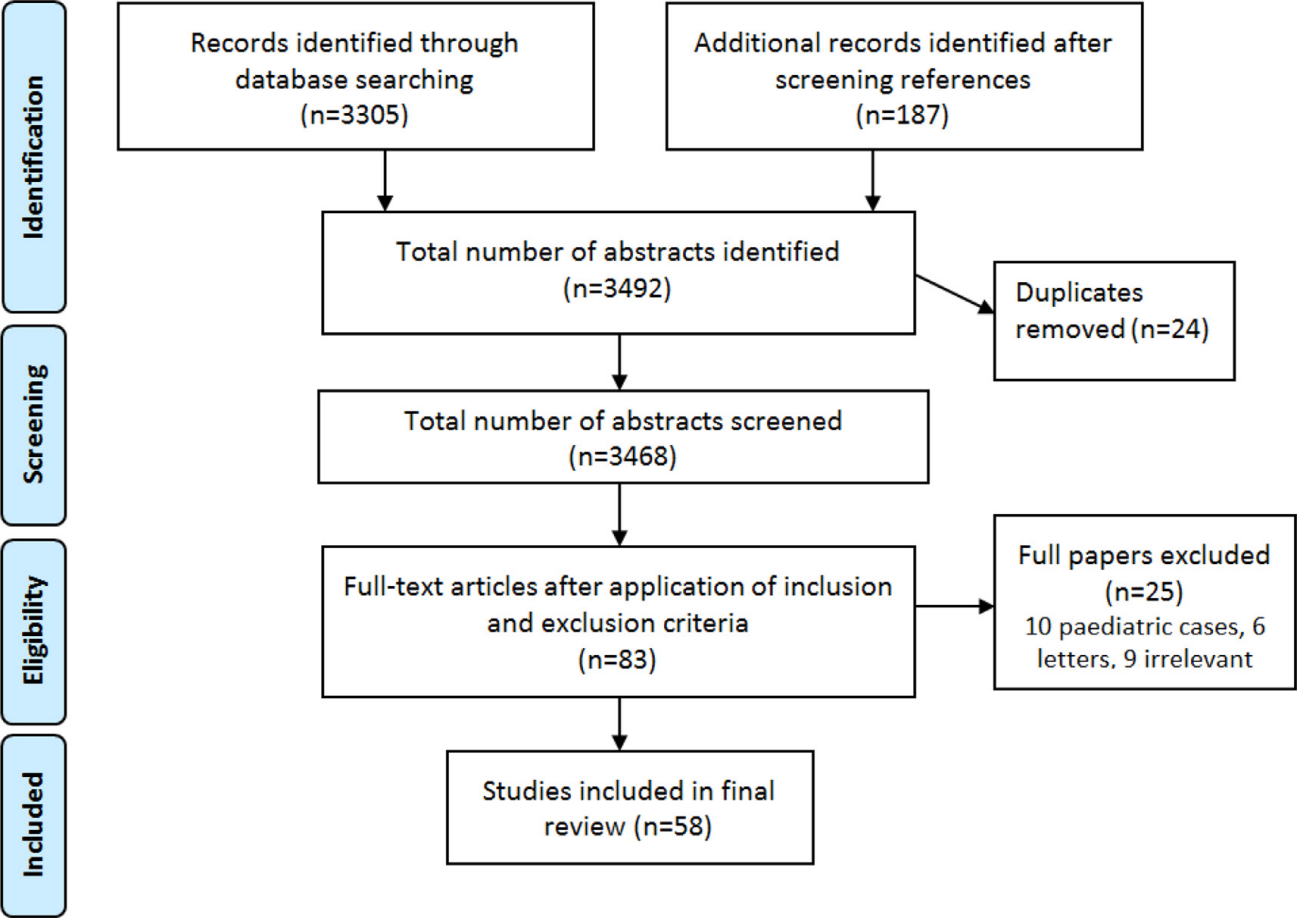
**PRISMA** flow diagram.

**Fig. 2:**
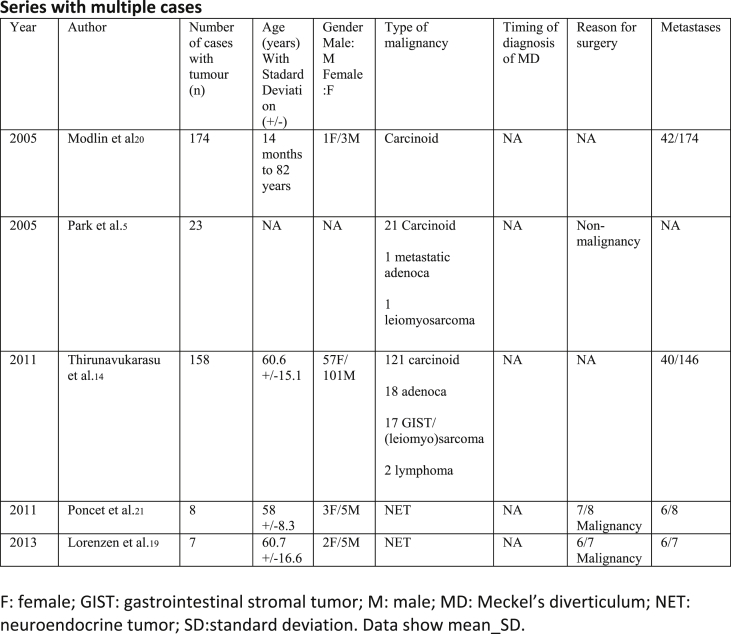
Systematic literature review.

**Fig. 3:**
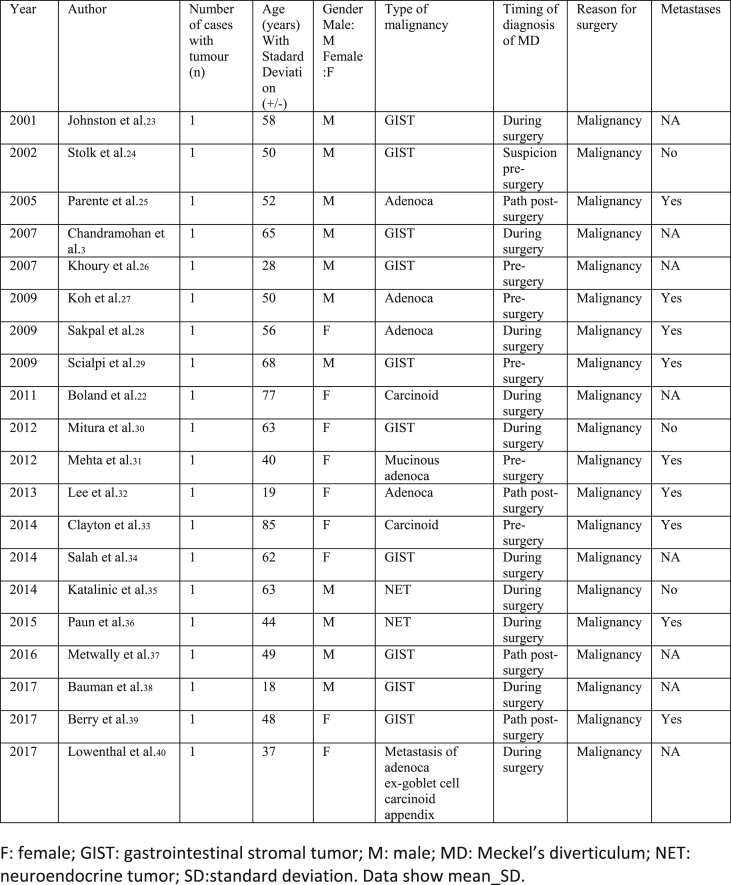
Single case reports with indication for surgery: malignancy.

**Fig. 4:**
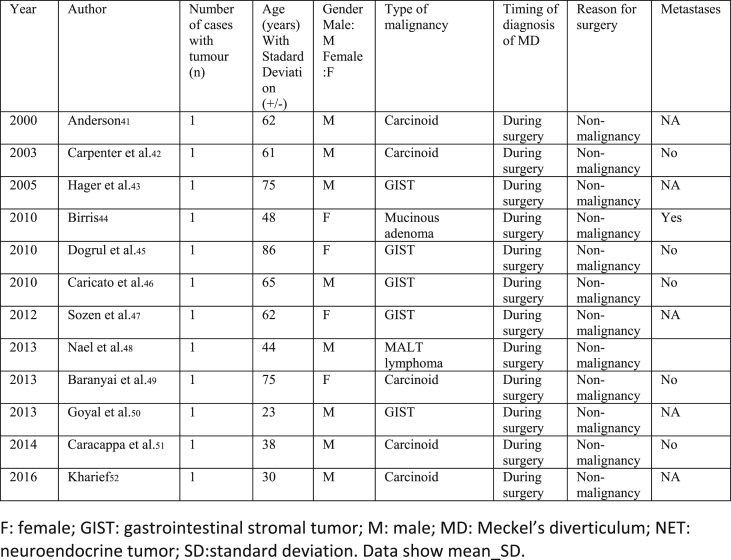
Single case reports with indication for surgery: of non-malignant disease.

Our selections were based on the PRISMA Flow methodology (Fig. 1). Our included studies comprised of randomized controlled trials, meta-analyses, systematic reviews, retrospective studies, case series and case reports (Fig. 2, Fig. 3, Fig. 4).

## 3 Carcinoid tumour of the Meckel's diverticulum

The term “Carcinoid” was first coined by Oberdorfer in 1907, who described it as a type of neoplasm whose benign characteristics distinguished it from a cancer [16].

It is the commonest type of primary tumour of the small intestine originating from the entero-chromaffin cells. It can occur in any anatomical region of the human body, but most commonly has been found to be in the appendix, with the ileum being the second most affected site, generally located to be in its last 60 cm. It secretes various hormones, the most important of which are substance P and serotonin. Carcinoid can exhibit malignant behavior but commonly shows low aggressiveness, being asymptomatic in 70%–80% of cases[17].

Symptoms of intestinal Carcinoid tumours can be intermittent abdominal pains, gastrointestinal bleeding and obstruction. Apart from these physical symptoms the manifestation of a typical Carcinoid syndrome can occur in 10%–20% of patients with acute episodes of skin flushing, diarrhea, asthma attacks, development of cardiac lesions and unfortunately in 45% of patients with massive liver metastasis. The non-specificity of symptoms, especially in the early phase results in delayed diagnosis, and as a result nearly half of the patients present with advanced disease with the average time between the onset of symptoms and the final diagnosis made has been reported to vary from 2 to 20 years [18,19].

Since both MD and Carcinoid tumours are uncommon clinical entities the occurrence of a Carcinoid tumour within a Meckel's diverticulum is even more rarer, Modlin et al. [20] have reported that approximately 0.48%–0.74% of all Carcinoids occur within the MD. The average age of presentation of a Carcinoid within MD is 55 years, with an incidence 2.5 times higher in men than women [21].

Until 1988, only 52 cases of Carcinoid arising from MD had been reported [22],and in 1997 a review by Sutton et al. identified further 111 cases [23]. At present, the Surveillance, Epidemiology, and End Results (SEER) Program of the National Cancer Institute, the authoritative source of information on incidence and survival of cancer in the US, has reported only 121 cases [24].

Moyan et al. have also suggested that Carcinoids localized in the appendix or in the colon show a less aggressive behavior than those originating from bronchus or small bowel [25].

The clinical presentation of a Carcinoid from MD is closely related to the disease stage i.e. lesions less than 10 mm with intact muscle layers are rarely symptomatic, whereas those with a more aggressive local characteristics are frequently associated with local and systemic signs and symptoms. In terms of metastasis, Moertel et al. [26] have shown that Carcinoid tumours of less then 1 cm have an incidence rate of 2% metastasis, whereas lesions between 1 and 2 cm metastasize in 50% of cases and those larger than 2 cm metastasize in 80% of cases. However, significant higher rates have been observed by Thompson [27], who reported an incidence of metastasis of 18% for lesions < 1 cm and 85% for lesions between 1 and 2 cm.

As mentioned earlier the liver is the most common site of metastasis, with approximately 30% reported 5-year survival in such patients; lung and bone metastases are less frequent, and metastases are twice as common in women than men, most likely due to hormonal factors. Carcinoid tumours of MD should also be considered aggressive owing to their potential of early metastases; hence some published studies advocate resection of the adjacent ileal segment and corresponding mesentery for tumours >0.5 cm [28].

Thirunavukarasu et al. recently published a review on the controversies surrounding elective resection of MD [29]. They focused on the relative risk of malignant transformation and analyzed epidemiology, incidence, stage at first diagnosis and survival in 163 cases of MD Carcinoids and 6214 cases originating from the ileum. The authors highlighted that despite the low incidence of MD their risk of malignant transformation remains high (1.44 per 10 million inhabitants). They argued that an estimated risk of MD malignant transformation of 70 times higher than all other ileal locations and increasing risk with age, incidental MD is best treated with surgical resection and primary anastamosis.

In support of the above simple MD excision is considered adequate by most of the studies in the case of lesions <10 mm in size [30,31]. whereas others argue it is sufficient only for lesions less than 5 mm [32]. For larger lesions, resection of the ileal tract and the corresponding mesentery is generally recommended. The presence of secondary lymphatic or hepatic dissemination is not considered as a contraindication to surgery, which should include the treatment of hepatic metastases [33].

Residual disease is managed via combination of chemotherapy and symptomatic inhibition therapy with Octreotide acetate. Five-year survival remains at 75% for patients with bowel-circumscribed disease, while for patients with lymphatic or hepatic involvement it decreases to 50% and 20%, respectively [34].

## 4 Leiomyosarcoma of the Meckel's diverticulum

Fried [35] reported the first case of a sarcoma arising in MD as fibro-myo-sarcoma in 1902.

Until 1991 only 59 cases of leiomyosarcoma have been reported [36]. There is no specific age distribution and it has been as reported in patients as young as 20 years and as old as 89 with no sex predilection [37,38].

The commonest symptom of Leiomyosarcoma arising from the MD is abdominal pain followed by intestinal bleeding, but it has also been reported to present with weight loss, abdominal mass, obstruction and perforation with peritonitis and sepsis [39].

As mentioned earlier in majority of cases of MD the diagnosis is made intra-operatively. However, mesenteric angiography may have a definite role in suspected cases of small bowel tumours.

Saadia et al., [40] in 1986 described the diagnostic role of mesenteric angiography in collected cases of leiomyosarcomas and reported hyper-vascularization, signs of necrosis and well-defined outlines in 95%, 25% & 65% of the cases respectively. The artery supplying the tumour was enlarged in 55% of the cases and a drainage vein was visible in 45% (hyper-vascularization and feeding vessels). Other investigations such as plain X-rays and barium studies were found to be unhelpful.

The histological differentiation between Leiomyoma (benign) and Leiomyosarcoma (malignant) has shown to be difficult, and the lesion may occasionally be confused with other tumours of neural, vascular or fibroblastic origin. Golden and Stout [41] have suggested that if there are two or more mitoses per high power field, the lesion is most likely to be malignant. On the other hand, few clinicians believe that the absence of mitosis does not exclude the possibility of malignancy. Advocating tumour size to predict malignancy, Starr [42] in his series found no benign tumour larger than 7x5x5 cm and no malignant lesion smaller than 2x2x1cm, hence, arguing that tumours greater than 7 cm may be malignant even in the absence of mitotic figures.

The usual mode of spread of intestinal Leiomyosacoma is by vascular embolisation, with the liver being the most commonly involved organ, followed by lungs and brain. Local spread and peritoneal seeding, with massive abdominal sarcomatosis as a cause of death have also been reported, however, lymphatic spread is rare [43].

In terms of management of such cancers, unfortunately there is no unanimous approach. Lee [44]has suggested that the tumour needs to be excised with at least 10 cm of normal bowel on either side including the adjacent mesentery. This approach is justifiable and acceptable to most clinicians, reason being the precise histological nature (benign or malignant) of the lesion at the time of resection is uncertain and the possibility of regional lymph node involvement does exist. It has been shown that there is little or no increase in morbidity between wide segmental and limited resection of the small bowel and neither chemotherapy nor radiotherapy confers any additional benefit. Finally, due to the rarity of the disease very little is known about the long-term prognosis.

In the Mayo Clinic study, 63 patients with Leiomyosarcoma of the jejunum or ileum were followed up for 25 years, of these 10 were alive and well, 4 had evidence of recurrent disease [45], had died from the initial tumour and 4 from another type of cancer. The presence of necrosis in the initial tumour was noted to be a bad prognostic sign.45 Starr and Dockerty have reported a 50% five-year survival after curative resection [46].

The most up-to-date series from Cleveland Clinic has identified three important factors for prognosis i.e. a long duration of symptoms, a tumour size of <9 cm in diameter, and the absence of lymphatic or distant metastases [47].

In conclusion, the diagnostic difficulties faced with this type of cancer most likely worsen the prognosis. In some reports the cancer is indolent and slow growing, hence the patient may survive for many years.

## 5 Adenocarcinoma of the Meckel's diverticulum

Adenocarcinoma of the MD is extremely rare with only 16 cases reported before 1963 and, in the Surveillance Epidemiology and End Results (SEER, 1973 AD to 2006 AD) program, in the United States (US) 18 people have been reported to have Adenocarcinoma arising from the MD [48].

It has been suggested that Adenocarcinoma of the Meckel's arise mainly from heterotopic tissue located within the diverticulum, this includes pancreatic tissue, duodenal, jejunal, colonic and gastric mucosa [49].

Factors that may contribute towards malignant degeneration of ectopic gastric mucosa remain disputed amongst clinicians, some speculate that ectopic gastric mucosa may have an increased malignant potential in comparison to normal bowel mucosa, where as others blame it on *Helicobacter pylori* (H. Pylori) as it is a well known carcinogen that has shown to be implicated in the pathogenesis of gastric Adenocarcinoma and mucosa-associated lymphoid tissue (MALT) lymphoma [50].

However, the role of H. Pylori in the pathogenesis of primary malignancy within the Meckel's diverticulum also remains questionable. Reiber et al. [51] has reported a case of synchronous gastric Adenocarcinoma with a second primary in the Meckel's diverticulum. They identified many *H. pylori* in the moderately differentiated Adenocarcinoma from the gastro-esophageal junction, but none in the neo-plastic tissue of the MD.

Symptoms and signs that can point towards neoplasm in a MD range from acute symptoms such as severe gastrointestinal bleeding or perforation, to chronic symptoms, such as obstruction and anemia. There have also been rare case reports about the coincidence of MD with intestinal mal-rotation in pediatric population. Ford et al. [52] reported the co-incidence of MD in up to 11% of children diagnosed with intestinal mal-rotation. In adult population there are only few reported cases of MD associated with intestinal mal-rotation, but no reported cases of malignant tumour in association with mal-rotation.

It remains extremely challenging to diagnose malignancies in a MD pre-operatively. The suspicion of it being malignant is often difficult at the initial stage and when malignancies are diagnosed, it is more likely to be at an advanced stage [53].

Based on the literature available to date, the treatment of a neoplasm within a MD typically involves diverticulectomy with primary small bowel anastomosis and an appendecectomy, with more extensive procedures individualized if additional disease or metastases are present. The role and benefit of adjuvant chemotherapy (5-fluorouracil, cisplatin, oxaliplatinin or mitomycin-C) is not clear, but its use has been reported in published literature [54,55].

## 6 Gastro intestinal stromal tumours (GISTs) of the Meckel's diverticulum

Gastro Intestinal Stromal Tumours (GISTs) arise from the interstitial cells of Cajal, also known as the pace maker cells of Gastro-intestinal tract. It occurs predominantly in adults at a median age of 58 years and accounts for 0.1–3% of all gastrointestinal neoplasms [56].

The definition of GIST has changed significantly since Mazur and Clarkto first introduced the term in 1983 [57]. Originally, it encompassed gastrointestinal non-epithelial neoplasms that lacked the immuno-histo-chemical features of Schwann cells and did not have the ultra-structural characteristics of smooth muscle cells. Therefore, based on this original classification of GIST, it has been reported that 42% of all tumours and 41% of malignant tumours of Meckel's diverticula would be classified as GIST [58].

Since GIST has now been accepted as a separate tumour entity and is defined as a spindle cell, epithelioid or pleo-morphic mesenchymal tumour of the gastrointestinal tract that strongly expresses the KIT (CD 117) protein and may harbour mutations of the type III tyrosine kinase receptor gene (either *KIT* or *PDGFRA*) [59].

In majority of patients symptoms tend to arise only when GIST reaches a significant size i.e. larger than 5 cm in maximal dimension or is in critical anatomic location. The symptoms include abdominal pain, abdominal mass, nausea, vomiting, anorexia, and weight loss. The vast majority of metastatic GISTs are located intra-abdominally, either in the liver, omentum, or in the peritoneal cavity [60]. Metastatic spread to lymph nodes and to other regions via lymphatics is very rare. CT is usually an adequate technology to diagnose GIST arising from MD [61].

In terms of prognosis there is very little data available for GISTs, and current prognostic indicators are based on consensus guidelines. The most important adverse factors are thought to be a tumour diameter of >5 cm and a high mitotic count exceeding five mitotic figures per 50 high powered fields on light microscopy. Other suggested factors indicative of poor prognosis include tumour perforation, tumour necrosis, high cellularity and marked pleomorphism [62,63].

Surgery is considered the standard treatment for non-metastatic GIST with en-bloc resection to obtain clear margins. The most recent data on GISTs presenting in the United States between 1992 and 2000 states a 5-year survival of 50–60% after complete resection of the localized primary tumour [64]. There is little evidence supporting local or regional lymphadenectomy as GISTs rarely metastasize to lymph nodes. Targeted therapy with Imantinib (KIT tyrosine kinase inhibitor) is considered the standard treatment for metastatic GIST [65].

## 7 Other rare tumours of the Meckel's diverticulum

Other tumours of the MD mentioned in the literature include pancreatic carcinoma, intra ductal papillary mucinous neoplasm, lymphomas and melanomas [[5], [6], [7], [8], [9], [10], [11], [12], [13]].

Unfortunately, due to the rarity of such histological sub-type tumours related to MD as mentioned above the literature has so far been limited to case reports only and what we can conclude form it is that, “the diagnosis is often delayed until the occurrence of a potentially life-threatening complications, such as intestinal intussusceptions, obstruction, bleeding or perforation have developed,” and is diagnosed at such an advanced stage commonly by either a CT or at laparotomy that there are limited treatment options available i.e. usually a surgical procedure is performed on an emergency basis with en bloc or de-bulking resection or other palliative procedures such as a bypass between non diseased terminal ileum and transverse colon is attempted followed by palliative chemotherapy and/or radiotherapy with very poor prognostic survival outcome [[5], [6], [7], [8], [9], [10], [11], [12], [13]].

## 8 Discussion

Primary malignancies of the MD are extremely rare with reported incidence to be only 0.5%–3.2%, and are usually diagnosed incidentally with poor overall prognosis dependent upon multiple factors such as age of the patient, metastasis, histological type/sub-type and biological aggressiveness of the neoplastic process, there is no formal pre or post grading and staging classification to date.

The prognosis is significantly worse and surgical resection futile if the primary malignant tumour remains covert or residual disease is present after surgery, adjuvant chemotherapy used appears to have no significant effect on its overall survival prognosis.

For better prognostic outcome it is important that the primary tumour of the MD is diagnosed early, and as MD is mostly discovered incidentally in an emergency setting the question to ask will be “Should an incidental MD be excised routinely to reduce the risk of developing into a tumour in the future?”

Previously, Soterro and Bill, based on there reported case series argued that an up to 800 incidental diverticulectomies are required in order to save one life, and the procedure itself has a complication rate of up to 8%, including a mortality rate of 1.2%. This outweighs the 2–4% lifetime risk of developing complications from Meckel's. Therefore, most authors did not advocate incidental diverticulectomy in every patient found to have a MD. However, more recently Dumper et al. [2] have recommend a case-by-case approach with factors favoring resection and reducing the risk of developing into a tumour such as younger age at presentation, palpable or visual abnormality of the Meckel's, previous symptoms which may be caused by the Meckel's such as obstruction or bleeding.

## 9 Conclusion

It is difficult to determine on any standard treatment, as malignant tumour in MD is a rare entity with unpredictable natural history that is usually discovered at an advanced stage during emergency, Unfortunately at that point limited surgical treatment options becomes available i.e. en bloc or de-bulking resection or other palliative procedures such as a bypass between non diseased terminal ileum and the transverse colon or an end ileostomy is attempted followed by palliative chemotherapy and/or radiotherapy with very poor prognostic survival outcome.

Overall prognosis is dependent upon multiple factors such as age of the patient, metastasis, histological type/sub-type and biological aggressiveness of the neoplastic process, at present there is no agreed standard formal grading and staging classification for primary MD tumour that can not only help assess the tumour in a systematic way, but also advise on a standard treatment plan that is to be followed after emergency surgery, that now needs to be developed.

## Provenance and peer review

Not commissioned, externally peer reviewed.

## Ethical approval

Not required.

## Sources of funding

N/A.

## Author contribution

S A Kabir - Study design, data collections, data analysis, writing.

S I Kabir - Study design, data collections, data analysis, writing.

S Raza - Data analysis.

## Conflicts of interest

No conflicts of interest.

## Research Registration number

UIN No: reviewregistry444.

http://www.researchregistry.com/browse-the-registry.html#registryofsystematicreviewsmeta-analyses/registryofsystematicreviewsmeta-analysesdetails/5a7a172d10da1267aa1f9180/

## Guarantor

N/A
